# Inhibition of Ehrlich ascites carcinoma growth by melatonin: Studies with micro-CT

**DOI:** 10.32604/or.2023.042350

**Published:** 2023-11-15

**Authors:** SEHER YILMAZ, ZÜLEYHA DOĞANYIĞIT, MERT OCAK, EVRIM SUNA ARIKAN SÖYLEMEZ, ASLI OKAN OFLAMAZ, SÜMEYYE UÇAR, ŞÜKRÜ ATEŞ, AMMAD AHMAD FAROOQI

**Affiliations:** 1 Department of Anatomy, Faculty of Medicine, Yozgat Bozok University, Yozgat, Turkey; 2 Department of Mechanical and Aerospace Engineering, Case Western Reserve University, Cleveland, Ohio, USA; 3 Department of Histology and Embriology, Faculty of Medicine, Yozgat Bozok University, Yozgat, Turkey; 4 Department of Anatomy, Faculty of Dentistry, Ankara University, Ankara, Turkey; 5 Department of Medical Biology, Faculty of Medicine, Afyonkarahisar Health Sciences University, Afyon, Turkey; 6 Department of Anatomy, Faculty of Medicine, Erciyes University, Kayseri, Turkey; 7 Department of Molecular Oncology, Institute of Biomedical and Genetic Engineering (IBGE), Islamabad, Pakistan

**Keywords:** Signaling pathway, Apoptosis, Real-time PCR, AgNOR

## Abstract

Melatonin is a versatile indolamine synthesized and secreted by the pineal gland in response to the photoperiodic information received by the retinohypothalamic signaling pathway. Melatonin has many benefits, such as organizing circadian rhythms and acting as a powerful hormone. We aimed to show the antitumor effects of melatonin in both *in vivo* and *in vitro* models through the mammalian target of rapamycin (mTOR) signaling pathway and the Argyrophilic Nucleolar Regulatory Region (AgNOR), using the Microcomputed Tomography (Micro CT). Ehrlich ascites carcinoma (EAC) cells were administered into the mice by subcutaneous injection. Animals with solid tumors were injected intraperitoneally with 50 and 100 mg/kg melatonin for 14 days. Volumetric measurements for the taken tumors were made with micro-CT imaging, immunohistochemistry (IHC), real-time polymerase chain reaction (PCR) and AgNOR. Statistically, the tumor tissue volume in the Tumor+100 mg/kg melatonin group was significantly lower than that in the other groups in the data obtained from micro-CT images. In the IHC analysis, the groups treated with Tumor+100 mg/kg melatonin were compared when the mTOR signaling pathway and factor 8 (F8) expression were compared with the control group. It was determined that there was a significant decrease (*p* < 0.05). Significant differences were found in the total AgNOR area/nuclear area (TAA/NA) ratio in the treatment groups (*p* < 0.05). Furthermore, there were significant differences between the amount of mTOR mRNA for the phosphatidylinositol 3-kinase (PI3K), AKT Serine/Threonine Kinase (PKB/AKT) genes (*p* < 0.05). Cell apoptosis was evaluated with Annexin V in an *in vitro* study with different doses of melatonin; It was observed that 100 µg/mL melatonin dose caused an increase in the apoptotic cell death. In this study, we have reported anti-tumor effects of melatonin in cell culture studies as well as in mice models. Comprehensive characterization of the melatonin-mediated cancer inhibitory effects will be valuable in advancing our fundamental molecular understanding and translatability of pre-clinical findings to earlier phases of clinical trials.

## Introduction

Cancer is a multifaceted disease and evidence gathered from proof-of-concept studies over decades of research has revolutionized our understanding about underlying causes of carcinogenesis and metastasis [1]. Tumor growth is known to occur in two distinct phases, known as early and late vascularization phases. The transformation of tumor development from the less damaging nonvascular stage to the more damaging vascular stage is an important cycle in cancer development, known as angiogenesis. Angiogenesis is known as a process in which new blood vessel networks are produced, unlike the existing vascular system, to meet the metabolic needs of the active tumor [2].

The PI3K/AKT/mTOR pathway in cell cycle regulation is one of the cellular signaling pathways widely known and reported to be associated with cancer. PI3Ks, AKT and mTOR are involved in the survival signaling of cancer cells [3]. For these reasons, increased proliferation and loss of apoptosis of cells are associated with cancer. PI3K/AKT/mTOR signaling network has different downstream effects on cellular functions. Deregulation of oncogenic signaling and metabolic reprogramming leads to cell survival and proliferation. Cancer cells are mostly activated by the AKT-mTOR pathway and this activation may adversely affect the course of the disease and its outcomes. It is known that 35% of breast cancer patients have PI3K mutations [4,5].

Various methods and substances have been used in the diagnosis and treatment of cancer. One of these methods is known as micro-CT. Micro-CT, recommended for imaging small animal disease models, micro-CT is a preclinical analog of clinical CT offering higher resolution (voxel size <=100 micron) [6,7]. To meet the desired requirements for higher resolution, micro-CT scanners use microfocused X-ray sources and operate at lower measured anode currents (50–1000 μA) and lower voltages (20–100 kVp) than clinical scanners. Unlike clinical CT scanners, which have curved detector arrays, micro-CT systems have smaller pixel sizes [8]. Studies have demonstrated that melatonin has oncostatic properties in different tumor types [9–11]. Contrary to popular belief, melatonin is known not only as a hormone but also as a cellular protective agent and has effects on immunomodulation, antioxidative processes and hematopoiesis. Melatonin has an oncostatic structure through different receptor-dependent and independent mechanisms [12,13]. Studies show that unregulated microRNAs (miRNAs) are conducive to cancer improvement and tumor growth. Melatonin limits the increase in tumors [14].

EAC is originally hyperdiploid, has high transplantability, no regression, rapid proliferation and mortality, and has no immunologically tumor-specific transplantation antigens. Nucleolar regulatory regions (NORs) consist of ribosomal DNA (rDNA) and have argiophilic properties [15].

Various studies have reported that melatonin reduces matrix metallopeptidase 9 (MMP-9) activation and proliferation [16,17]. However, to the best of our knowledge, the effects of different doses of melatonin on the mTOR signaling pathway in EAC cells have not yet been investigated with different imaging methods. This study aimed to determine the anticarcinogenic effect of melatonin, which was applied *in vivo* and *in vitro* at different doses on EAC cells, by micro-CT measurements and its effect on the mTOR signaling pathway.

## Materials and Methods

### Experimental model design

**Figure 1:**
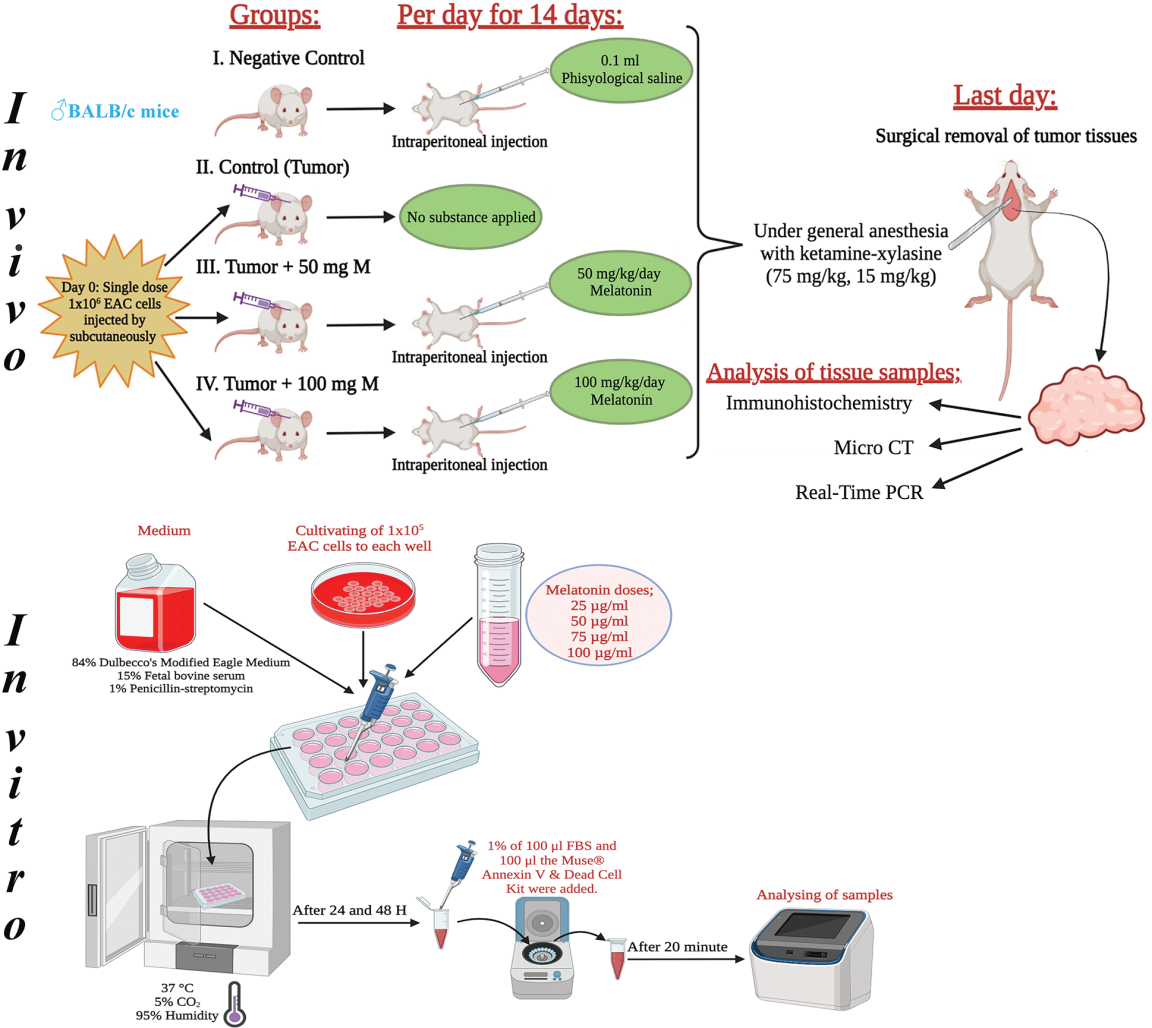
Schematic view of the design of the *in vivo* and *in vitro* experiments. The figure was created using the free trial version of Biorender (Biorender.com).

This research included 8- to 10-week-old, 25- to 30-g male BALB/c mice. It used 40 male mice in total, with 10 mice in each group. Melatonin was diluted with physiological saline and the doses determined in the experiment were administered intraperitoneally to the experimental animals in the dark cycle throughout the experiment, taking into account the circadian rhythm. Fig. 1 shows the design of the *in vivo* and *in vitro* experiments. The tumor volume (mm^3^) = width × height × 0.52 formula was used when calculating the tumor volumes [18].

### Reagents and antibodies

Melatonin (SLBN2691V, Sigma Aldrich, Product of China), silver nitrate (Cas no: 7761-88-8, Wertheim/Germany), PureZole reagent (Bio-Rad, USA, Cat no: 732-6890), iScript Reverse Transcription Supermix (Bio-Rad, USA, Cat no: 1708891), SYBR Green Supermix (Bio-Rad, USA, Cat no: 172-5270), mTOR (sc-517464, Santa Cruz Biotechnology, Inc., Texas, USA), PI3K (sc-1637, Santa Cruz Biotechnology, Inc., Texas, USA) and AKT (60203-2-Ig, Proteintech, Germany) protein expression was determined by immunohistochemistry.

### Creation of stock cells

The cells, which were kept at −80 degrees before, were thawed at room temperature and 0.1 cc i.p. was given to the animal, which was determined as a stock. Tumor formation was noticed in the stock animal within 6–7 days. Acid tumor cell fluid collected from EAT cells counted on a Thoma slide was injected by subcutaneous injection into mice identified at 8–10 weeks of age to form solid tumors.

### Micro-CT procedure

Percent tissue volume refers to the ratio of the total volume (TV) of available tissue to the objective volume (OV) analyzed. It is a commonly used parameter in some pathologies that reflects object gain/loss. It indicates the fraction of a given volume of interest occupied by tissue. Tumor tissues from the experiment were scanned with a micro-CT device (SkyScan 1275, Kontich, Belgium). The resulting configuration and parameters are as follows: 1 mm aluminum filter, 80 keV, 125 μA, 10 μm resolution, exposure = 47 ms, rotation = 180 and rotation step = 0.400. Reconstruction data were obtained using NRecon reconstruction software (version 1.6.9.4; Bruker Corp., Billerica, MA, USA). All experimental measurements were evaluated with CTAN software (version 1.17.7.2; Bruker Corp., Billerica, MA, USA), and the resulting 3D images were created using the CTVol software (version 2.3; Bruker Corp., Billerica, MA, USA).

### Histological analysis

Histological analysis was performed as described in a previous study. Ehrlich ascites carcinoma cells were examined under a light microscope [19].

### Immunohistochemical analysis

Immunohistochemical analysis was performed as described in a previous study by Doğanyiğit et al. [19].

### AgNOR staining

AgNORs was done on the basis of the protocol established by the Quantitative Committee reported in previous studies by Ploton et al. [20].

### Genetic analysis

RNA was extracted using PureZole reagent (Bio-Rad, CA, USA). All RNA samples were reverse transcribed using the iScript Reverse Transcription Supermix (Bio-Rad, USA). Expression analysis of the mTOR signaling pathway was performed using Rotor Gene-Q (Qiagen, Hilden, Germany) and SsoAdvanced SYBR Green Supermix (Bio-Rad, USA). PI3K-F: 5’-AACACAGAAGACCAATACTC-3’, PI3K-R: 5’-TTCGCCATCTACCACTAC-3’, Akt1-F: 5’GTGGCAAGATGTGTATGAG-3’, Akt1-R: 5’CTGGCTGAGTAGGAGAAC-3’, Mtor-F: 5’-GACAACAGCCAGGGCCGCAT-3’, Mtor-R: 5’-ACGCTGCCTTTCTCGACGGC-3’, Gapdh-F: 5’-GAGGACCAGGTTGTCTCCTG-3’, Gapdh-R: 5’-GGATGGAATTGTGAGGGAGA-3’.

### Cell culture

Cell culture analysis was performed as described in a previous study by Ateş et al. [21].

### Statistical analysis

Data from the experiment were statistically evaluated using the GraphPad Prism program (version 8.0, San Diego, California) and presented as the mean value ± SD. One-way analysis of variance (ANOVA) was used to analyze the data obtained and Dunnett’s multiple comparison test was used as a *post hoc* test in the follow-up. A value of *p* < 0.05 was considered statistically significant.

## Results

### Tumor volume findings

When the tumor volumes in the experimental groups were compared on the 9th, 11th, 13th and 14th days, there was a statistically significant difference between the groups in terms of tumor volumes (*p* < 0.05). Table 1 shows the data for this comparison.

**Table 1:** Daily changes in tumor volumes

Day	Control (tumor) median (25%–75%)	Tumor+50 mg/kg melatonin median (25%‒75%)	Tumor+100 mg/kg melatonin median (25%‒75%)	*p*
7	394.0 (195.1–562.7)	-	-	-
8	667.7 (328.9–1060)	355.0 (158.5–420.9)	351.1 (214.4–439.4)	0.0716
9	1932 (543.0–2590)^a^	467.3 (279.9–602.0)^b^	624.7 (379.3–841.6)^b^	0.0180
10	2515 (703.0–2223)	727.8 (552.4–763.0)	1197 (427.3–1474)	0.1872
11	1834 (1111–2749)^a^	852.7 (595.6–1167)^b^	1095 (535.4–1242)^ab^	0.0378
12	5414 (1596–3382)	1001 (547.7–1575)	1375 (794.8–1636)	0.2077
13	3040 (2053–3674)^a^	954.7 (486.9–1055)^b^	1441 (722.0–2206)^b^	0.0017
14	3253 (2157–3665)^a^	1093 (600.7–1206)^b^	1291 (584.5–2121)^b^	0.0019

Note: Volumetric changes in the tumors belonging to the experimental groups. Values measured in mm^3^. Data are presented as the mean ± SD (standard deviation). ^a, b^*p* < 0.05 was considered statistically significant.

### Micro-CT findings

**Figure 2:**
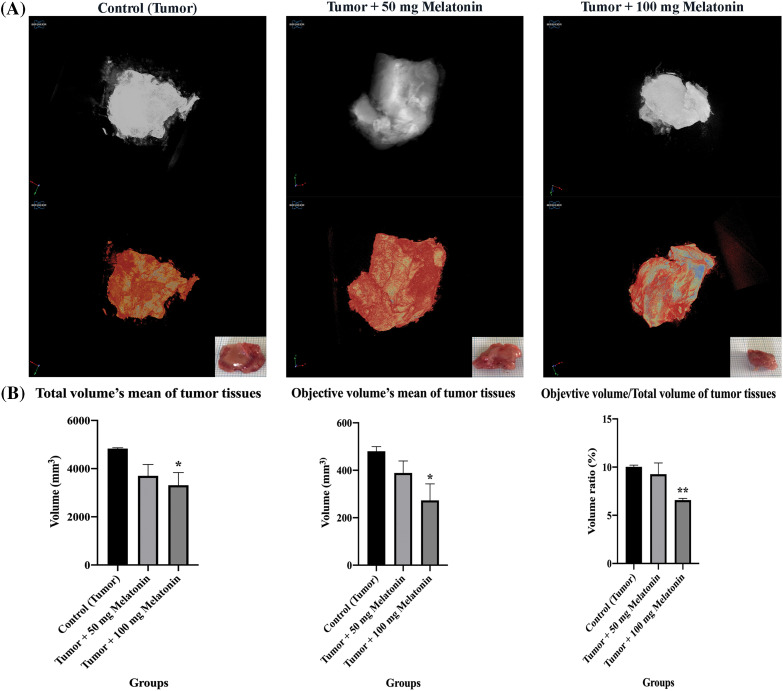
(A) *Ex vivo* tumor tissue images of experimental groups taken with micro-CT. (B) Tissue volume (OV/TV), total volume of tissue present relative to the analyzed objective volume (OV). The data are presented as mean ± SD. ANOVA *p < 0.05, **p < 0.01. The figure was created using the photoshop software (version 23.3.2).

Tumor tissues were visualized with micro-CT in the present study. There was a statistically significant decrease in tissue volume percent objective volume/total volume (OV/TV) in the Tumor+100 mg/kg melatonin group (Fig. 2).

### Histological findings

**Figure 3:**
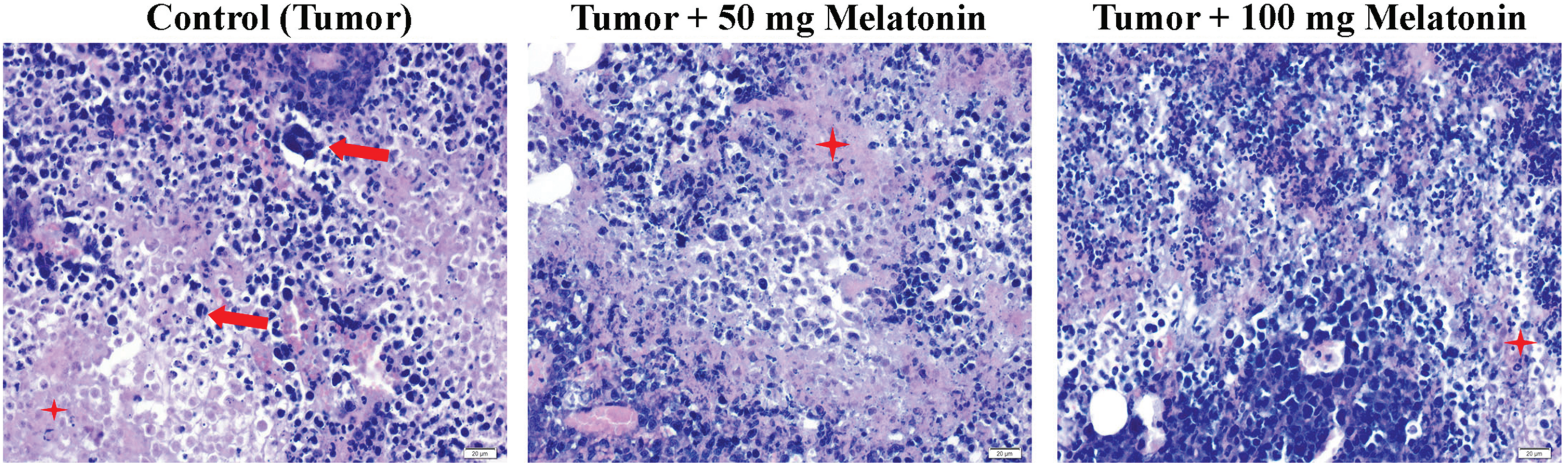
Hematoxylin and eosin staining images from tumor tissues of the experimental groups. The red arrow indicates Ehrlich’s hyperchromatic large-nucleated carcinoma cells and the red star indicates eosinophilic cytoplasm. Magnification: 40X, bar = 20 μm. The figure was created on photoshop (version 23.3.2).

The Control (Tumor) group had hyperchromatic and large-nucleated cells of different shapes and sizes and eosinophilic dense cytoplasm. Eosinophilic cytoplasm and hyperchromatic large nuclei were observed less frequently in the Tumor+100 mg/kg melatonin group (Fig. 3).

### Immunohistochemistry analysis findings

**Figure 4:**
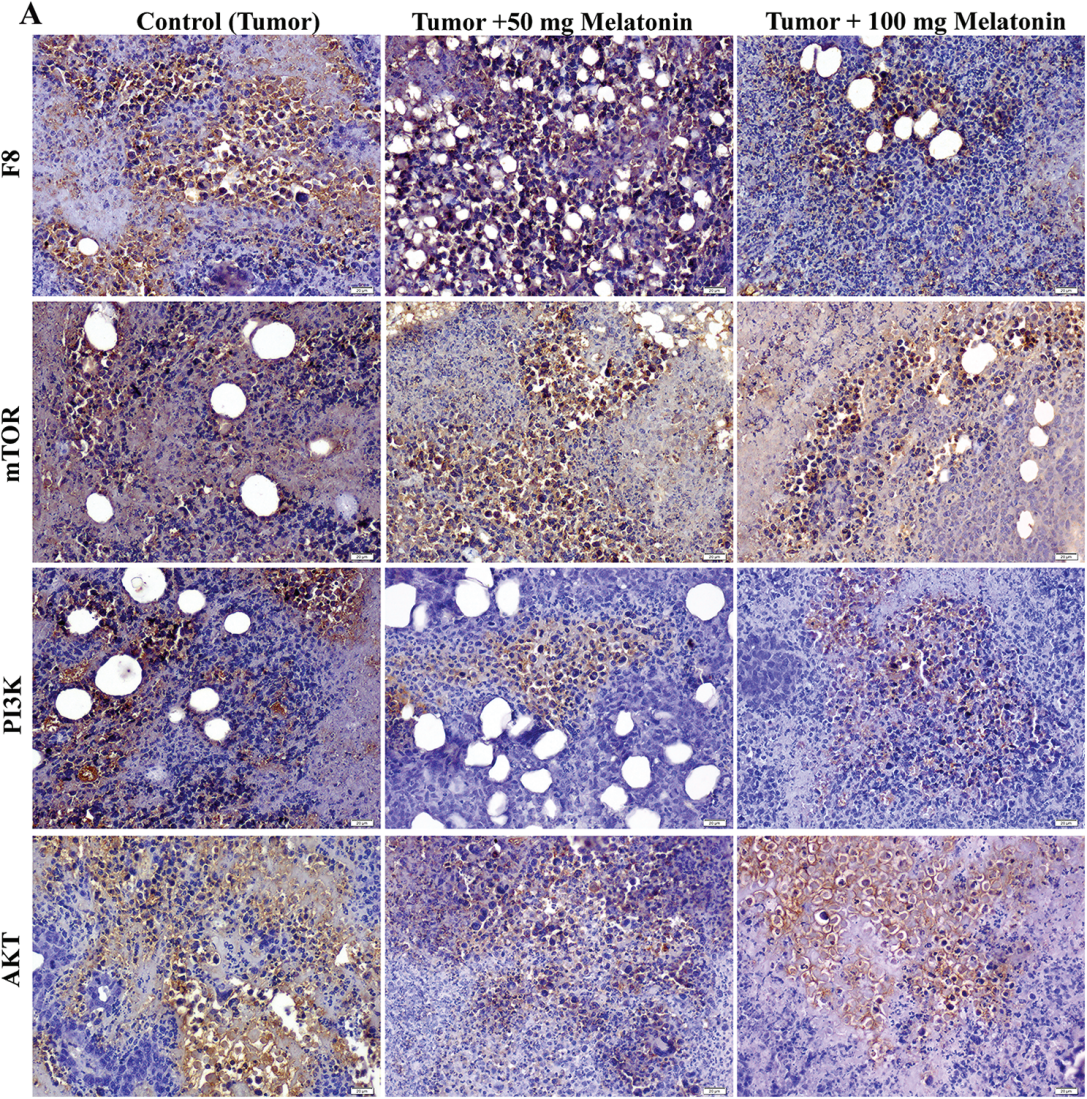
Images of the effects of melatonin use on F8, AKT, mTOR and PI3K expression in tumor sections of the experimental groups. Magnification: 40X, bar = 20 μm. The figure was created on photoshop (version 23.3.2).

**Figure 5:**
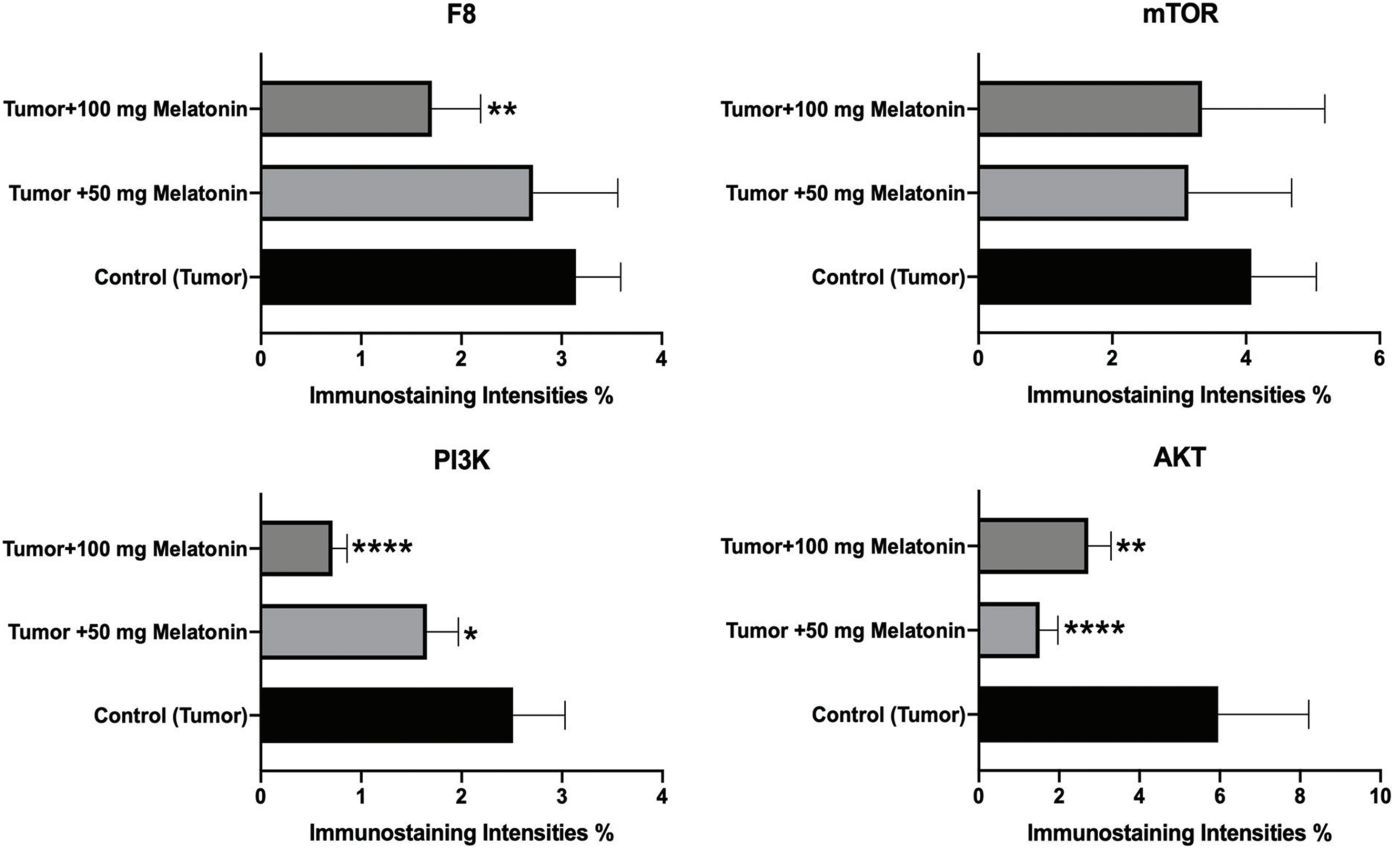
Histogram plots showing F8, AKT, mTOR and PI3K immunostaining intensities. The values indicated in the histogram are expressed as mean ± SD. The ANOVA and Dunnett’s multiple analysis were applied to the graph. *p < 0.05, **p < 0.01 and ****p < 0.0001 show the difference in the control (tumor) group. The figure was created on photoshop (version 23.3.2).

According to the data obtained, there was no significant difference between the groups in terms of mTOR expression. F8 expression was found to be considerably reduced in the Tumor+100 mg/kg melatonin-administered groups. This decrease was found to be 1.47 times. PI3K and AKT protein expression was significantly decreased compared to that in the Control (Tumor) groups. Importantly, PI3K expression was reduced to a greater extent in the Tumor+100 mg/kg melatonin-treated group, whereas, AKT expression was markedly reduced in the tumor and 50 mg/kg melatonin-treated group. PI3K expression decreased 1.51 times in the Tumor+50 mg/kg melatonin group compared to the Control (Tumor) group and 3.51 times in the Tumor+100 mg/kg melatonin group. Compared to the Control (Tumor) group, the expression of AKT decreased 3.92-fold in the Tumor+50 mg/kg melatonin group, while it decreased 2.18 times in the Tumor+100 mg/kg melatonin group (Figs. 4 and 5).

### AgNOR staining findings

**Figure 6:**
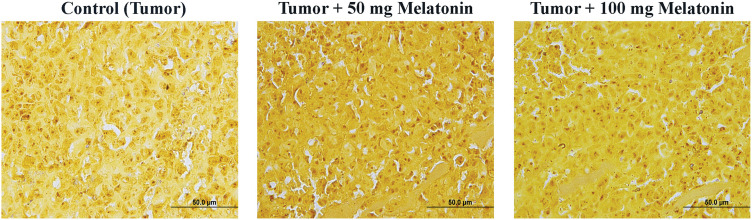
AgNOR-stained cells in the experimental groups. AgNOR: argyrophilic-nucleolar organizer region. Magnification: 40X, bar = 50 μm. The figure was created on photoshop (version 23.3.2).

Cells stained with AgNOR are shown in Fig. 6. Significantly different values were observed between the experimental groups in terms of the TAA/NA ratio (x2 = 62,411, *p* = 0.000) and mean AgNOR number (x2 = 108,250, *p* = 0.000) (Table 2).

**Table 2:** AgNOR and TAA/NA counts in the three groups

Groups	TAA/NA	AgNOR number	*p*	x2
Control (tumor)	0.26 ± 0.06	6.79 ± 1.39	0.000*	62.411*
Tumor+50 mg/kg melatonin	0.22 ± 0.04	5.04 ± 1.56	0.000	
Tumor+100 mg/kg melatonin	0.16 ± 0.02	2.31 ± 0.42	0.000	108.250

Note: Data are expressed as mean ± SD. *p < 0.05 was taken as statistically significant.

### Gene expression findings

**Figure 7:**
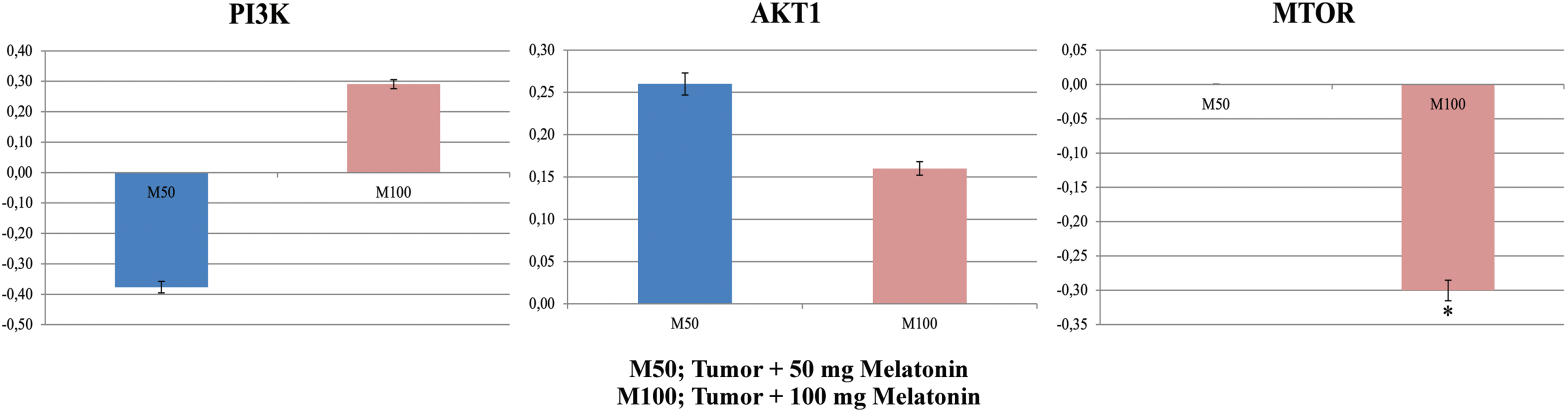
In experimental groups administered 50 and 100 mg/kg melatonin, mRNA expression levels and fold regulation levels of PI3K, AKT1, and mTOR are shown. GAPDH was used as the reference gene. Data are expressed as mean ± SD. *p < 0.001. The figure was created on photoshop (version 23.3.2).

The PI3K gene mRNA expression level reduced in the Tumor+50 mg/kg melatonin group compared to the Control (Tumor) group. PI3K gene mRNA levels were increased in the Tumor+100 mg/kg melatonin group compared to the Control (Tumor) group (*p* > 0.05). AKT1 gene mRNA levels were increased in the Tumor+50 mg/kg melatonin and Tumor+100 mg/kg melatonin groups compared to the Control (Tumor) group (1.83 and 1.44-fold, respectively) (*p* > 0.05). The mTOR gene mRNA level was not changed (1.00-fold) in the Tumor+50 mg/kg melatonin group and the mTOR gene mRNA level was decreased in the Tumor+100 mg/kg melatonin group compared to the Control (Tumor) group (0.5-fold, *p* < 0.001) (Fig. 7).

### In vitro findings

**Figure 8:**
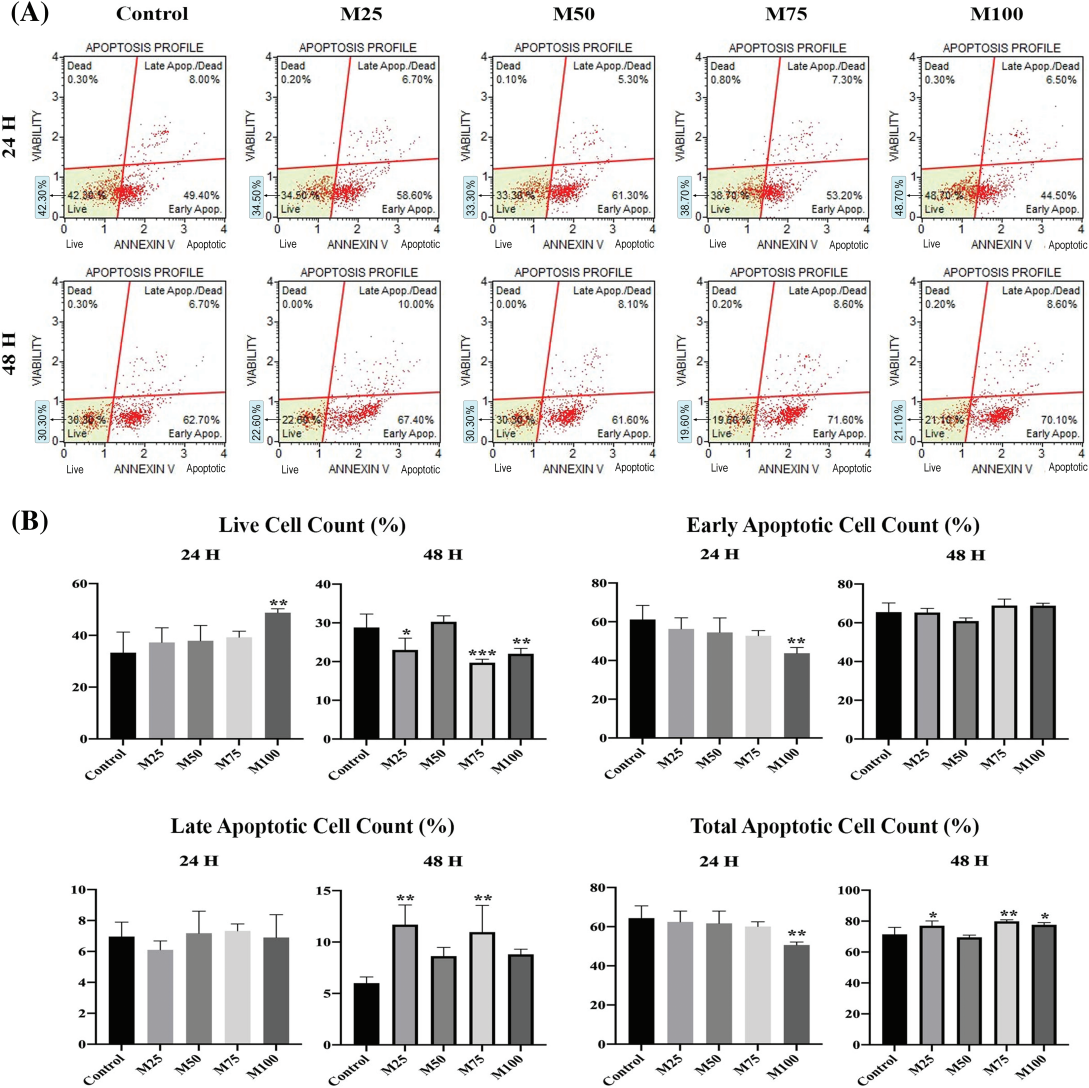
(A) Raw graph from the muse cell analyzer (Annexin V and apoptosis profile). (B) Annexin V & dead cell assay measurements after 24 and 48 h. *p < 0.05, **p < 0.005, and ***p < 0.001 were considered statistically significant compared to the control. Data are expressed as mean ± SD. Control group: Control, melatonin groups: M25, M50, M75, M100. The figure was created on photoshop (version 23.3.2).

Graphs from the Muse Cell Analyzer (Fig. 8A). EAC cell survival and early, late and total apoptosis were calculated. In the analyses, cell viability was measured in the early period and late period. There was a significant increase in the data acquired from the Muse Cell Analyzer when the live cells were determined (*p* < 0.005) in the M100 group compared to the control group at the end of the 24-h incubation. At the end of the 48-h incubation, the live cell count was lower in M25 (*p* < 0.05), M75 (*p* < 0.001) and M100 (*p* < 0.005) (Fig. 8B).

## Discussion

Cancer cells can permanently evade the immune system by using diverse mechanisms, including activation of various systems [22]. The tumor progression process is the result of a combination of several different steps. In this process, malignant tumors may escape from many local or systemic regulatory mechanisms and become immune-resistant and drug-resistant. In this process, cancer cells autoregulate the behavior of normal cells for the growth of cancer tissue [23]. The main role of melatonin in the activation of the anticancer immune response has been discussed in various studies. Because melatonin is known as a powerful antioxidant, it directly inactivates free radicals and produces active metabolites. In addition to the known pleiotropic effects of melatonin and some of its metabolites, mitochondrial redox and homeostasis effects have also been reported [24,25]. However, melatonin is reported to induce apoptosis against many cancer types currently being investigated [26,27]. *In vitro* and *in vivo* studies involving both experimental animal models and clinical trials are available in the literature to evaluate the features of melatonin as a single agent or as an adjunct to antitumor strategies. Seminal studies also provide proof-of-concept for the determination of melatonin doses [28]. One study reported that the survival rate increased due to the use of melatonin and the response rates were positively accelerated when melatonin was used in addition to several antitumor treatments. In addition, melatonin alleviates the toxic and side effects of chemotherapy and reduces the symptoms associated with malignancy [29]. Recently, under the conditions of acute acidosis in breast cancer cell lines, apoptosis has been induced and cell proliferation decreased in melatonin-treated groups. Studies have shown that pretreatment with melatonin before ionizing radiation causes a reduction in cell proliferation and an increase in p53 mRNA expression levels, leading to increased radiosensitivity in breast cancer cells [30]. We established a solid tumor model of EAC in an experimental mouse model and determined the antioxidant properties of different doses of melatonin on solid tumors *in vivo* and *in vitro*. We also demonstrated that melatonin significantly inhibited the activity of the mTOR signaling pathway. The phosphatidylinositol-3-kinase (PI3K)/Akt and mammalian rapamycin target (mTOR) signaling pathways are both very important for cell growth and survival in both physiological and pathological situations. The PI3K/Akt signaling pathway is a key organ of survival during cellular stress [31]. The role of signaling pathways in cancer is important, as tumors proliferate and are inherently in a stressful environment. Mutations of certain genes, gains or losses due to these mutations, are among a series of genetic changes that affect these pathways found in a number of different solid and hematological tumors. The activation of the PI3K/Akt/mTOR pathway causes a profound disruption in the control of cell growth and survival, ultimately leading to a competitive growth advantage, metastatic competence, angiogenesis, and therapy resistance [32]. Since we did not perform knockdown or inhibitor experiments in our study, even if Melatonin inhibits PI3K, Akt and mTOR expressions, this does not mean that these factors mediate the anticancer effect. However, it has been reported that melatonin induces apoptosis of cancer cells by suppressing PI3K, Akt and mTOR signaling pathways [33]. Yilmaz et al. reported in their study that the antioxidant rutin, which they administered at different doses, decreased the tumor volume by suppressing the mTOR signaling pathway [34]. During the growth phase of the tumor, the increase and invasion of tumor cells gradually increases. Melatonin inhibits the progression of cancer cells by downregulating MMP-9 and fibroblast growth factor 19 to inhibit the invasion and migration of cancer cells [35]. In addition, *in vivo* studies have shown that melatonin slows down the progression of cancer by deactivating the Notch homolog 1 (Notch 1) receptor [33,36]. Combined treatment with rapamycin and melatonin blocks the negative feedback loop from the specific downstream effector of mTOR activation S6K1 to Akt signaling, which reduces cell viability, proliferation and clonogenic capacity [37]. One study examined the effects of oral melatonin supplementation on the growth of intraperitoneally implanted EAC cells in female mice. The dose of melatonin was adjusted to 50 mg/kg, and the dose of melatonin administered decreased the viability and volume of Ehrlich acid carcinoma cells and was reported to increase the survival rate of treated mice. They stated that glutathione-S-transferase activity increased significantly in EAC cells and that melatonin not only delayed the progression of cells from the G(0)/G(1) phase of the cell cycle to the S phase but also reduced DNA synthesis during the cell cycle [38]. A study examined the antineoplastic properties of prodigiosins in the Ehrlich solid tumor model, indicating that prodigiosins stopped the cell cycle in the G2/M phase, decreased the percentage of cells in the S phase and prevented cell proliferation [39]. AgNOR staining has been proposed as a useful tool for the diagnosis and prognosis of cancer. One study examined AgNOR staining profiles obtained with protein extracts from Ehrlich tumor cell nucleoli isolated by a new procedure that preserves nucleolar fine structure on western blots. They showed by immunostaining that the polypeptide could be associated with the nucleolar phosphoprotein pp135, which was demonstrated in rat cell nucleoli [40]. Nisari et al. administered certain doses of curcumin to mice carrying EAC and showed that curcumin has a very important function against cancer development as an AgNOR biomarker. They reported that the amount of protein decreased due to exposure to curcumin [41]. Yılmaz et al. examined the effects of quercetin on EAC-carrying mice with AgNOR staining and reported that there was an improvement in the groups given quercetin compared to the control by using the mean AgNOR number and TAA/NA ratio in routine cytopathology [15]. The micro-CT is capable of creating high-resolution 3D images at the cellular level. Resolution at the cellular level is likely to increase soon, as the existing theoretical limits of X-ray imaging are greater than those of optical imaging and as micro-CT imaging technology is advancing day by day. In their study, Spiro et al. compared micro-CT with clinical CT and clinical MRI regarding the monitoring of treatment effects in mice with lung cancer and found that micro-CT could be an appropriate method for evaluating the response to treatment in mice with cancer [42].

## Conclusion

Melatonin anticancer activity is stimulated by interfering with various cancer features through different signaling pathways. In our study, we observed and demonstrated the effects of melatonin on EAC with micro-CT and on the mTOR signaling pathway. In our experiment, melatonin administered at different doses *in vivo* and *in vitro* caused apoptosis in tumor cells. The effects of melatonin on the signaling pathway were evaluated *in vivo* and *in vitro*, supported by TAA/NA and genetic analysis. Furthermore, micro-CT imaging methods in cancer can be used as an alternative method in cancer examination in terms of diagnostic examinations.

## Data Availability

All data included in this article can be obtained from corresponding author upon reasonable requirements.

## Abbreviations

mTOR: Mammlian target of rapamycin
Micro-CT: Microcomputed tomography
EAC: Ehrlich ascites carcinoma
PCR: Polymerase chain reaction
AKT: Serine/Threonine kinase (PKB/AKT)
PI3K: Phosphatidylinositol 3-kinase
F8: Factor 8
AgNOR: Argyrophilic nucleolar organizer region
TAA: Total AgNOR area
NA: Nuclear area
